# Quantifying the Impact of Obesity Category on Major Chronic Diseases in Canada

**DOI:** 10.1100/tsw.2007.216

**Published:** 2007-08-17

**Authors:** Ying Jiang, Yue Chen, Douglas Manuel, Howard Morrison, Yang Mao, Obesity Working Group

**Affiliations:** ^1^ Centre for Chronic Disease Prevention and Control, Public Health Agency of Canada, 120 Colonnade Road, Locator 6702A, Ottawa, Ontario, Canada K1A 0K9,; ^2^ Department of Epidemiology and Community Medicine, Faculty of Medicine, University of Ottawa, 451 Smyth Road, Ottawa, Ontario, Canada K1H 8M5,; ^3^ Institute for Clinical Evaluative Sciences, 2075 Bayview Avenue, Toronto, Ontario, Canada M4N 3M5,

**Keywords:** body mass index, obesity, prevalence, logistic regression model, population attributable risk

## Abstract

Adverse health effects differ with various levels of obesity, but limited national data existed previously for the Canadian population. We examined the associations of sociodemographic and behavioral factors with obesity levels in Canada, and measured the impact of each level on major chronic diseases. Data were extracted from the 2003 Canadian Community Health Survey. We grouped overweight/obese participants aged 18 years and over into four levels based on body mass index (BMI, kg/m^2^): overweight (25.0– 29.9), class I obesity (30.0–34.9), class II obesity (35–39.9), and class III obesity (extreme/clinical obesity, BMI ≥ 40.0). We used logistic regression models to identify potential risk factors for the obesity levels and to estimate adjusted odds ratios (ORs) for major chronic diseases related to each level. We calculated population attributable risks (PARs) to help understand the impact of obesity levels on these chronic diseases.

The overall prevalence of obesity was 16.2% in men and 14.6% in women, and the prevalence of obesity III was 1.0% in men and 1.4% in women. All levels of obesity increased with age, but then decreased in elderly participants. The prevalence of diabetes, hypertension, heart disease, arthritis, and asthma increased with increasing BMI level, and the highest values appeared in participants at the obesity III level. PAR was highest in the obesity III group for hypertension, followed by diabetes, and lowest for heart disease. When correlated with risk factors, fewer statistically significant ORs, comparing to the normal weight category, appeared for obesity II and III levels than for overweight and obesity I. ORs for the combination of low education level, infrequent exercise, and low household income rose significantly with BMI levels until the obesity II level, and in obesity III level, the OR remained at the same level as for obesity II, most significantly in women. These results suggest that the impact of obesity on Canadian’s health should be studied and dealt with by obesity level. The greatest impact of clinical obesity was on hypertension and diabetes control in Canada.

## INTRODUCTION

Obesity has been increasing in Canada for several decades[1,2,3]. From 1970–2004, obesity prevalence rose 8–23% in men and 13–22% in women, and the increase occurred in all age groups[4,5]. Most epidemiological studies of obesity show that morbidity and mortality increase among populations with body mass index (BMI) above 30 $kg/m^2$[6,7]. The population attributable fraction for all-cause mortality and selected major chronic diseases associated with obesity increased from 1970 to 2004 by 60% for men and by 37% for women[5]. Overall, the estimated economic burden of obesity in Canada in 2001 was $4.3 billion, and the health care costs of obesity represented 2.2% of total health care expenditures[8].

Health risks for individuals rise progressively with increasing degree of obesity. Obesity has been categorized as class I (BMI = 30.0–34.9), class II (BMI = 35.0–39.9), or class III (BMI ≥ 40) according to differences in treatment and health risks[7,9]. Persons with class III obesity (considered as *clinical obesity*) have been shown to have substantial increases in risk of morbidity and mortality[7,10]. The odds of incurring any health care expenditure are much greater among adults with class III obesity[11].

Generally speaking, obesity is a major determinant for many chronic diseases, such as hypertension, diabetes mellitus, cardiovascular disease, stroke, and gallstones[12]. In Canada, however, limited national data existed on the association of different categories of overweight and obesity, especially of clinical obesity, with the major chronic diseases. The objectives of this study were to describe the prevalence of obesity by levels in persons aged 18 years and over in Canada, to examine the association of sociodemographic and health behavior factors with different obesity levels, and to measure the impact of obesity levels on the prevalence of major chronic diseases. The estimates produced may provide baseline figures for developing and managing a nationwide obesity action plan.

## METHODS

### Data Source

We used data from the Canadian Community Health Survey (CCHS, Cycle 2.1), collected between January and December 2003 for 126 health regions, from all provinces and territories. The CCHS 2.1 sampling frame covered approximately 98% of the Canadian population aged 12 and over, with 127,610 participants. We restricted our study to participants aged 18 years and over[13].

### Variables

We calculated BMI by dividing the weight in kilograms by the square of the height in meters ($kg/m^2$). We grouped participants into BMI levels according to the classification system identified by the International Obesity Task Force: underweight, BMI < 18.5; normal weight, BMI = 18.5–24.9; overweight, BMI = 25.0–29.9; class I obesity, BMI = 30.0–34.9; class II obesity, BMI = 35.0–39.9; and class III obesity (clinical obesity), BMI ≥ 40.0[12]. BMI was not calculated for pregnant women. Additionally, we excluded 381 men and 3519 women who were not asked to provide information on body weight and height.

Based on previous research findings, we included the following covariates in the association analysis: age, race, language spoken most often at home, marital status, urban/rural location, education level, physical activity, income, type of smoker, and frequency of drinking alcohol. There were four age categories: 18–34, 35–49, 50–64, and 65 years and over. Exercise was defined as physical activity lasting more than 15 min. We considered regular exercise to be physical activity performed monthly at least 12 times; occasional exercise, 4–11 times; and infrequent exercise, 0–3 times. Participants were classified into low-, middle-, and high-income groups based on total annual household income adjusted for number of household members[14]. Other variables included smoking (current, exsmoker, nonsmoker), race

(white, Aboriginal, others), language spoken most often at home (English, French, others), marital status (married/common-law, single, others), location (urban, rural), education level (less than university, university degree or more), and alcohol drinking (yes, no).

We included five chronic diseases (hypertension, diabetes mellitus, heart disease, arthritis, and asthma) in the analysis. Disease data were based on self-reported positive responses to the question "Do you have hypertension, diabetes mellitus, heart disease, arthritis, or asthma diagnosed by a health professional?"[15,16]

## Analysis

All analyses were performed separately for men and women. We calculated the prevalence of health outcomes, disease determinants, and risk factors. Odds Ratios (ORs) were calculated for each obesity level vs. the normal BMI level (18.5–24.9). All estimates were weighted to the general Canadian population. We estimated 95% confidence intervals (CIs) by using a bootstrap approach to account for the complex survey design.

We generated multivariate logistic regression models to assess the associations between obesity levels and potential independent sociodemographic and health behavior predictors. We also conducted multivariate logistic regression analyses to estimate the adjusted ORs for hypertension, diabetes mellitus, heart disease, arthritis, and asthma among participants in the various BMI levels, controlling for covariates. In addition, we calculated ORs by BMI level for respondents with low education level, infrequent exercise, and low household income, relative to those with high education level, frequent exercise, and medium or high household income, after adjusting for age, race, language spoken, location, marital status, smoking status, and alcohol drinking.

We calculated the population attributable risks (PARs) and attributable numbers from adjusted ORs, using the following equations:

$$PAR = p(OR-1) / (p(OR-1) + 1)$$

where p is the probability of a patient being obese in a given patient population and OR is the odds ratio for the specific chronic disease of an obese subject.

$$\text{Attributable number} = PAR^{*} N$$

where N is the number of obese persons in the general population.

All statistical analyses were performed using the SAS software system for Windows, version 8.

## RESULTS

This analysis included 110,224 participants aged 18 years or over living in Canada in 2003. Of the 51,153 men, 16.2% were classified as obese (1.0% in class III), as were 14.6% of the 59,071 women (1.4% in class III). Overweight was more common than obesity in both men and women across all variables (Table 1). The prevalence of overweight and obesity I was higher in men than in women, whereas the prevalence of obesity II and III was lower in men. The prevalence of obesity in Aboriginal participants was significantly higher than in other racial groups for men at obesity level I–II and for women at obesity level I–III.

As depicted in Table 2, the prevalence of diabetes, hypertension, heart disease, arthritis, and asthma was higher in obese men and women.

**TABLE 1**
**Weighted Descriptive Statistics (Percentage* and 95% CI†) for Overweight and Obese Men and Women Aged 18 Years or Older, by BMI Category, CCHS 2003, Canada**

| Sex | Variable | BMI (kg/m$^2$) | | | |
|---|---|---|---|---|---|
| | | 25.0–29.9 | 30.0–34.9 | 35.0–39.9 | ≥40 |
| Male | Age (years) | | | | |
| | 18–34 | 33.0 (31.8, 34.3) | 9.9 (9.2, 10.6) | 2.5 (2.1, 2.8) | 0.8 (0.6, 1.1) |
| | 35–49 | 44.9 (43.7, 46.2) | 12.7 (12.0, 13.4) | 2.8 (2.4, 3.1) | 0.9 (0.7, 1.1) |
| | 50–64 | 45.6 (44.1, 47.0) | 15.9 (15.0, 16.9) | 3.2 (2.6, 3.9) | 1.5 (1.0, 2.1) |
| | 65+ | 44.1 (42.6, 45.6) | 11.9 (10.9, 12.8) | 2.1 (1.6, 2.5) | 0.4 (0.3, 0.6) |
| | All ages | 414 (40.7, 42.0) | 12.5 (12.1, 12.9) | 2.7 (2.4, 2.9) | 1.0 (0.8, 1.1) |
| | Marital status | | | | |
| | Married/common-law | 45.0 (44.1, 45.8) | 13.8 (13.2, 14.3) | 2.8 (2.5, 3.1) | 1.0 (0.8, 1.2) |
| | Single | 31.7 (30.3, 33.0) | 9.0 (8.2, 9.7) | 2.3 (1.9, 2.7) | 0.9 (0.6, 1.2) |
| | Widow/separated/divorced | 41.7 (39.7, 43.6) | 12.9 (11.6, 14.1) | 2.5 (1.9, 3.0) | 1.0 (0.6, 1.4) |
| | Location | | | | |
| | Urban | 40.6 (39.8, 41.4) | 11.9 (11.4, 12.4) | 2.5 (2.2, 2.7) | 0.9 (0.8, 1.1) |
| | Rural | 44.5 (43.2, 45.8) | 14.9 (14.0, 15.7) | 3.4 (3.0, 3.9) | 1.0 (0.8, 1.3) |
| | Race | | | | |
| | White | 42.5 (41.9, 43.3) | 13.4 (13.0, 13.9) | 2.8 (2.6, 3.1) | 0.9 (0.8, 1.1) |
| | Aboriginal | 40.1 (34.0, 46.2) | 18.0 (14.0, 22.0) | 9.4 (5.1, 13.8) | 1.2 (0.5, 2.0) |
| | Others | 34.2 (31.9, 36.4) | 7.3 (6.3, 8.4) | 1.0 (0.7, 1.4) | 0.8 (0.2, 1.4) |
| | Language spoken | | | | |
| | English | 42.6 (41.8, 43.3) | 13.8 (13.3, 14.3) | 3.0 (2.7, 3.2) | 0.9 (0.8, 1.1) |
| | French | 40.8 (39.1, 42.5) | 11.1 (10.1, 12.1) | 2.4 (1.9, 2.8) | 1.0 (0.6, 1.5) |
| | Others | 35.9 (33.2, 38.5) | 8.2 (6.7, 9.6) | 1.3 (0.9, 1.8) | 0.7 (0.1, 1.2) |
| | Education level | | | | |
| | < University | 40.3 (39.2, 41.3) | 13.8 (13.1, 14.5) | 3.2 (2.8, 3.5) | 1.1 (0.9, 1.3) |
| | ≥ University | 42.0 (41.1, 42.9) | 11.6 (11.0, 12.2) | 2.4 (2.1, 2.7) | 0.9 (0.7, 1.1) |
| | Income level | | | | |
| | High | 43.2 (42.4, 44.0) | 12.9 (12.4, 13.4) | 2.6 (2.3, 2.8) | 0.8 (0.7, 1.0) |
| | Medium | 38.0 (36.2, 39.8) | 12.3 (11.3, 13.4) | 2.8 (2.3, 3.3) | 1.0 (0.5, 1.4) |
| | Low | 33.3 (30.8, 35.8) | 11.8 (10.3, 13.3) | 3.3 (2.5, 4.1) | 1.8 (0.9, 2.8) |
| | Smoking status | | | | |
| | Nonsmokers | 39.3 (37.9, 40.6) | 10.8 (10.0, 11.6) | 2.6 (2.2, 2.9) | 1.3 (0.9, 1.7) |
| | Exsmokers | 45.5 (44.5, 46.5) | 14.1 (13.4, 14.7) | 2.9 (2.6, 3.3) | 1.0 (0.8, 1.2) |
| | Current smokers | 36.2 (34.9, 37.6) | 11.5 (10.7, 12.3) | 2.3 (1.9, 2.6) | 0.6 (0.4, 0.8) |
| | Alcohol drinking | | | | |
| | Yes | 42.2 (41.3, 43.2) | 13.0 (12.4, 13.6) | 2.7 (2.4, 3.0) | 0.7 (0.5, 0.9) |
| | No | 41.7 (40.5, 42.9) | 11.8 (11.1, 12.4) | 2.4 (2.1, 2.7) | 0.9 (0.7, 1.1) |
| | Physical activity | | | | |
| | Regular | 42.7 (41.9, 43.6) | 12.0 (11.5, 12.5) | 2.4 (2.1, 2.6) | 0.7 (0.5, 0.9) |
| | Occasional | 41.2 (39.5, 42.8) | 13.6 (12.5, 14.8) | 3.4 (2.8, 4.1) | 1.0 (0.7, 1.2) |
| | Infrequent | 37.1 (35.4, 38.8) | 13.4 (12.3, 14.4) | 3.2 (2.6, 3.7) | 1.7 (1.2, 2.3) |

**TABLE 1 (continued)**

| Sex | Variable | BMI (kg/m$^2$) | | | |
|---|---|---|---|---|---|
| | | 25.0–29.9 | 30.0–34.9 | 35.0–39.9 | ≥40 |
| Female | Age (years) | | | | |
| | 18–34 | 17.8 (16.9, 18.7) | 6.7 (6.1, 7.3) | 2.1 (1.8, 2.4) | 1.3 (0.9, 1.6) |
| | 35–49 | 25.2 (24.0, 26.4) | 9.8 (9.0, 10.6) | 3.2 (2.7, 3.6) | 1.4 (1.1, 1.6) |
| | 50–64 | 33.7 (32.5, 34.9) | 13.7 (12.8, 14.6) | 4.1 (3.7, 4.6) | 2.0 (1.7, 2.4) |
| | 65+ | 34.8 (33.7, 36.0) | 11.9 (11.1, 12.7) | 2.5 (2.1, 2.9) | 1.1 (0.8, 1.4) |
| | All ages | 26.8 (26.2, 27.4) | 10.2 (9.8, 10.6) | 3.0 (2.8, 3.2) | 1.4 (1.3, 1.6) |
| | Marital status | | | | |
| | Married/common-law | 28.6 (27.9, 29.4) | 10.8 (10.2, 11.3) | 3.0 (2.8, 3.3) | 1.4 (1.2, 1.6) |
| | Single | 17.8 (16.7, 18.8) | 7.2 (6.5, 8.0) | 2.5 (2.1, 2.9) | 1.6 (1.1, 2.0) |
| | Widow/separated/divorced | 30.8 (29.5, 32.0) | 11.6 (10.6, 12.5) | 3.3 (2.8, 3.7) | 1.4 (1.0, 1.8) |
| | Location | | | | |
| | Urban | 26.1 (25.4, 26.7) | 9.8 (9.4, 10.2) | 2.8 (2.6, 3.1) | 1.4 (1.2, 1.6) |
| | Rural | 30.3 (29.1, 31.4) | 12.0 (11.2, 12.8) | 3.6 (3.2, 4.0) | 1.5 (1.3, 1.8) |
| | Race | | | | |
| | White | 27.6 (27.0, 28.2) | 10.8 (10.3, 11.2) | 3.1 (2.9, 3.3) | 1.6 (1.4, 1.7) |
| | Aboriginal | 29.4 (24.8, 34.0) | 18.4 (14.5, 22.3) | 7.8 (5.3, 10.4) | 3.6 (2.1, 5.1) |
| | Others | 22.9 (21.0, 24.7) | 6.3 (5.1, 7.5) | 1.6 (1.1, 2.1) | 0.6 (0.3, 0.9) |
| | Language spoken | | | | |
| | English | 27.5 (26.9, 28.1) | 10.7 (10.3, 11.1) | 3.3 (3.0, 3.5) | 1.5 (1.3, 1.7) |
| | French | 26.6 (25.5, 27.7) | 9.9 (9.0, 10.8) | 2.5 (2.1, 3.0) | 1.6 (1.2, 2.1) |
| | Others | 24.5 (22.4, 26.6) | 8.0 (6.6, 9.4) | 2.0 (1.3, 2.7) | 0.8 (0.4, 1.2) |
| | Education level | | | | |
| | < University | 30.5 (29.5, 31.5) | 12.3 (11.6, 12.9) | 3.5 (3.1, 3.9) | 1.8 (1.5, 2.1) |
| | ≥ University | 24.4 (23.7, 25.1) | 8.8 (8.3, 9.3) | 2.6 (2.4, 2.8) | 1.2 (1.0, 1.4) |
| | Income level | | | | |
| | High | 26.0 (25.2, 26.7) | 9.6 (9.1, 10.1) | 2.7 (2.4, 2.9) | 1.3 (1.1, 1.6) |
| | Medium | 30.0 (28.7, 31.4) | 11.8 (10.8, 12.8) | 3.6 (3.1, 4.1) | 1.7 (1.3, 2.1) |
| | Low | 27.2 (25.5, 28.9) | 11.7 (10.5, 12.9) | 4.3 (3.5, 5.0) | 2.1 (1.7, 2.5) |
| | Smoking status | | | | |
| | Nonsmokers | 26.2 (25.3, 27.2) | 9.8 (9.1, 10.4) | 2.8 (2.5, 3.1) | 1.3 (1.1, 1.6) |
| | Exsmokers | 28.6 (27.6, 29.5) | 11.2 (10.5, 11.8) | 3.4 (3.1, 3.8) | 1.7 (1.5, 1.9) |
| | Current smokers | 24.7 (23.5, 25.8) | 9.3 (8.5, 10.1) | 2.5 (2.1, 2.8) | 1.2 (0.8, 1.5) |
| | Alcohol drinking | | | | |
| | Yes | 23.1 (22.1, 24.2) | 8.4 (7.7, 9.1) | 2.4 (2.1, 2.8) | 0.9 (0.7, 1.0) |
| | No | 28.2 (27.3, 29.0) | 10.1 (9.5, 10.6) | 3.0 (2.7, 3.2) | 1.6 (1.4, 1.9) |
| | Physical activity | | | | |
| | Regular | 26.3 (25.6, 27.0) | 9.1 (8.7, 9.6) | 2.5 (2.3, 2.8) | 1.1 (0.9, 1.3) |
| | Occasional | 26.8 (25.3, 28.3) | 10.9 (9.8, 11.9) | 3.5 (2.9, 4.0) | 1.7 (1.3, 2.0) |
| | Infrequent | 28.4 (27.0, 29.7) | 13.2 (12.2, 14.2) | 4.1 (3.6, 4.6) | 2.4 (1.9, 2.9) |

* The percentage estimates were weighted to the general Canadian population.

† Bootstrap method was used for 95% CI calculation.

**TABLE 2**
**Prevalence* of Selected Major Chronic Diseases by Sex and BMI Category, CCHS 2003, Canada**

| Disease | | BMI (kg/m²) | | | | | | | | | |
|---|---|---|---|---|---|---|---|---|---|---|---|
| | | 18.5–24.9 | | 25.0–29.9 | | 30.0–34.9 | | 35.0–39.9 | | ≥40 | |
| | | N | % | N | % | N | % | N | % | N | % |
| Men | | | | | | | | | | | |
| Asthma | Yes | 1220 | 6.1 | 1325 | 6.2 | 533 | 7.9 | 165 | 9.9 | 62 | 14.9 |
| | No | 18,722 | 93.8 | 20,026 | 93.8 | 6647 | 92.0 | 1454 | 90.1 | 458 | 85.1 |
| Arthritis | Yes | 2961 | 10.6 | 4155 | 15.1 | 1650 | 18.4 | 441 | 23.4 | 154 | 29.2 |
| | No | 16,966 | 89.4 | 17,171 | 84.8 | 5523 | 81.6 | 1176 | 76.5 | 365 | 70.8 |
| Hypertension | Yes | 2227 | 8.9 | 4065 | 16.8 | 1931 | 23.7 | 529 | 31.4 | 182 | 41.7 |
| | No | 17,689 | 91.1 | 17,233 | 83.2 | 5226 | 76.3 | 1087 | 68.5 | 337 | 58.2 |
| Diabetes | Yes | 768 | 3.0 | 1455 | 5.5 | 813 | 9.9 | 296 | 15.3 | 117 | 23.0 |
| | No | 19,182 | 96.9 | 19,891 | 94.5 | 6362 | 90.0 | 1322 | 84.6 | 403 | 77.0 |
| Heart disease | Yes | 1376 | 4.9 | 1756 | 6.2 | 678 | 8.0 | 182 | 9.1 | 44 | 11.1 |
| | No | 18,568 | 95.0 | 19,579 | 93.7 | 6496 | 92.0 | 1436 | 90.8 | 476 | 88.9 |
| Women | | | | | | | | | | | |
| Asthma | Yes | 2439 | 8.0 | 1691 | 9.3 | 926 | 12.1 | 377 | 19.3 | 195 | 20.2 |
| | No | 27,219 | 91.9 | 15,611 | 90.6 | 5937 | 87.9 | 1758 | 80.7 | 824 | 79.8 |
| Arthritis | Yes | 6684 | 17.4 | 5973 | 29.3 | 2768 | 34.9 | 875 | 37.0 | 417 | 38.4 |
| | No | 22,954 | 82.6 | 11,314 | 70.7 | 4087 | 65.1 | 1258 | 62.9 | 600 | 61.6 |
| Hypertension | Yes | 4284 | 10.6 | 4855 | 24.0 | 2432 | 30.3 | 789 | 33.8 | 397 | 35.4 |
| | No | 25,361 | 89.3 | 12,438 | 75.9 | 4423 | 69.6 | 1342 | 66.1 | 621 | 64.5 |
| Diabetes | Yes | 855 | 2.2 | 1254 | 6.0 | 875 | 11.0 | 342 | 15.3 | 209 | 17.4 |
| | No | 28,805 | 97.8 | 16,045 | 94.0 | 5986 | 89.0 | 1790 | 84.7 | 809 | 82.5 |
| Heart disease | Yes | 1710 | 4.0 | 1411 | 6.1 | 642 | 7.8 | 167 | 5.6 | 98 | 7.8 |
| | No | 27,932 | 95.9 | 15,881 | 93.8 | 6212 | 92.2 | 1964 | 94.3 | 919 | 92.2 |

* The percentage estimates were weighted to the general Canadian population.

Table 3 shows the adjusted ORs for sociodemographic and health behavior risk factors in different BMI levels by sex. Based on the statistically significant ORs, age, education level, and physical activity were major risk factors across BMI levels in both sexes. Among men, marital status, residential location, race, and language spoken were positively or negatively associated with overweight, obesity I, and obesity II, but not with obesity III. Alcohol drinking and exsmoking in men were associated with overweight and obesity I, but not with obesity II or III. Among women, marital status and alcohol drinking were not associated with obesity I or II, but with obesity III. The residential location of women was associated with overweight and obesity I, but not with obesity II or III.

Table 4 presents the adjusted ORs that we modeled for each obesity level and PARs for major chronic diseases in the different BMI categories. The trends of association were consistent across the five diseases. The adjusted odds of having one of these chronic diseases increased with increasing BMI levels, similar to the pattern observed in the PARs.

Respondents with a particular cluster of risk factors — low education level, infrequent exercise, and low household income — were compared with those with the opposite cluster (high education level, frequent exercise, and "non-low" household income) (Table 5). For each level of overweight/obesity, the adjusted ORs in women were higher than those in men. The ORs rose with BMI levels until the obesity II level and then remained constant at the obesity III level. This trend was more significant in women than in men.

**TABLE 3**
**Logistic Regression Model for Each Obese Category (ORs) and Potential Sociodemographic and Health Behavior Risk Factors, CCHS 2003, Canada**

| Risk Factor | BMI (kg/m$^2$) | | | | | | | |
|---|---|---|---|---|---|---|---|---|
| | Men | | | | Women | | | |
| | 25–29.9 | 30–34.9 | 35–39.9 | ≥40 | 25–29.9 | 30–34.9 | 35–39.9 | ≥40 |
| Age (years) | | | | | | | | |
| 18–34 | ref | ref | ref | ref | ref | ref | ref | ref |
| 35–49 | 1.5* | 1.4* | 1.2 | 1.3 | 1.4* | 1.5* | 1.7* | 1.1 |
| 50–64 | 1.7* | 1.8* | 1.5* | 2.2* | 2.3* | 2.4* | 2.5* | 1.8* |
| 65 + | 1.3* | 1.0 | 0.6* | 0.3* | 2.1* | 1.6* | 1.0 | 0.6* |
| Marital status | | | | | | | | |
| Married/common-law | ref | ref | ref | ref | ref | ref | ref | ref |
| Single | 0.6* | 0.5* | 0.6* | 0.8 | 0.8* | 0.9 | 1.1 | 1.4* |
| Widow/separated/divorced | 0.8* | 0.8* | 0.7* | 0.9 | 0.9* | 0.9 | 1.0 | 0.9 |
| Location | | | | | | | | |
| Rural | ref | ref | ref | ref | ref | ref | ref | ref |
| Urban | 0.9* | 0.8* | 0.8* | 1.0 | 0.9* | 0.9* | 0.9 | 1.0 |
| Race | | | | | | | | |
| White | ref | ref | ref | ref | ref | ref | ref | ref |
| Aboriginal | 1.5* | 1.9* | 4.3* | 1.8 | 1.7* | 2.3* | 3.1* | 2.9* |
| Others | 0.7* | 0.5* | 0.3* | 0.6 | 0.8* | 0.5* | 0.4* | 0.3* |
| Language spoken | | | | | | | | |
| English | ref | ref | ref | ref | ref | ref | ref | ref |
| French | 0.8* | 0.7* | 0.7* | 0.9 | 0.9* | 0.8* | 0.6* | 0.9 |
| Others | 0.8* | 0.7* | 0.6* | 0.5* | 0.9 | 0.8* | 0.7 | 0.6 |
| Education level | | | | | | | | |
| ≥ University | ref | ref | ref | ref | ref | ref | ref | ref |
| < University | 1.1* | 1.3* | 1.5* | 1.4* | 1.3* | 1.4* | 1.4* | 1.5* |
| Income | | | | | | | | |
| High | ref | ref | ref | ref | ref | ref | ref | ref |
| Medium | 0.9* | 1.0 | 1.1 | 1.1 | 1.2* | 1.3* | 1.6* | 1.4* |
| Low | 0.8* | 1.0 | 1.4* | 1.9* | 1.2* | 1.3* | 1.9* | 1.7* |
| Physical activity | | | | | | | | |
| Regular | ref | ref | ref | ref | ref | ref | ref | ref |
| Occasional | 1.0 | 1.2* | 1.5* | 1.5* | 1.1* | 1.3* | 1.6* | 1.8* |
| Infrequent | 0.9* | 1.1 | 1.4* | 2.5* | 1.2* | 1.6* | 2.0* | 2.9* |
| Smoking status | | | | | | | | |
| Nonsmokers | ref | ref | ref | ref | ref | ref | ref | ref |
| Exsmokers | 1.2* | 1.2* | 1.1 | 0.9 | 1.1 | 1.1 | 1.2 | 1.2 |
| Current smokers | 0.8* | 0.8* | 0.6* | 0.3* | 0.9 | 0.8* | 0.6* | 0.6* |
| Alcohol drinking | | | | | | | | |
| No | ref | ref | ref | ref | ref | ref | ref | ref |
| Yes | 1.3* | 1.3* | 1.2 | 1.0 | 1.0 | 0.9 | 0.8 | 0.5* |

* ORs are statistically significant ($p < 0.05$).

**TABLE 4**
**Adjusted ORs and PARs and Their 95% CIs for Major Chronic Diseases Associated with Different BMI Levels by Sex, CCHS 2003, Canada⁺**

| Disease | BMI Category | | | | | | | |
|---|---|---|---|---|---|---|---|---|
| | Men | | | | Women | | | |
| | Overweight | Obesity I | Obesity II | Obesity III | Overweight | Obesity I | Obesity II | Obesity III |
| Asthma | | | | | | | | |
| OR (95% CI)* | 1.1 (0.9, 1.2) | 1.4 (1.2, 1.7) | 1.7 (1.3, 2.3) | 2.8 (1.5, 5.1) | 1.2 (1.1, 1.4) | 1.6 (1.4, 1.8) | 2.6 (2.1, 3.3) | 2.7 (2.0, 3.6) |
| PAR % (95% CI) | 0.6 (0, 1.2) | 3.1 (1.6, 5.2) | 6.5 (2.9, 11.4) | 21.1 (6.9, 37.9) | 1.8 (0.9, 3.6) | 6.8 (4.6, 8.8) | 23.6 (17.5, 30.7) | 25.6 (16.8, 34.4) |
| AN† | 1 790 | 3 559 | 2 015 | 3 535 | 5 210 | 9 722 | 15 584 | 8 572 |
| Arthritis | | | | | | | | |
| OR (95% CI) | 1.3 (1.2, 1.4) | 1.6 (1.4, 1.8) | 2.4 (1.9, 3.0) | 3.6 (2.4, 5.4) | 1.4 (1.3, 1.5) | 1.9 (1.7, 2.1) | 2.4 (2.0, 2.8) | 2.8 (2.2, 3.6) |
| PAR % (95% CI) | 4.3 (2.9, 5.7) | 9.9 (6.9, 12.8) | 24.7 (17.4, 31.9) | 43.2 (29.0, 56.2) | 10.5 (8.1, 12.8) | 23.9 (19.6, 27.7) | 34.1 (27.0, 40.0) | 40.9 (31.5, 50.0) |
| AN | 31 400 | 26 615 | 18 114 | 14 105 | 95 289 | 98 148 | 43 207 | 25 884 |
| Hypertension | | | | | | | | |
| OR (95% CI) | 1.8 (1.6, 2.0) | 2.9 (2.6, 3.4) | 5.3 (4.2, 6.7) | 8.6 (5.8, 12.7) | 2.0 (1.8, 2.2) | 3.0 (2.7, 3.4) | 4.4 (3.6, 5.4) | 5.1 (3.9, 6.8) |
| PAR % (95% CI) | 11.8 (9.2, 14.4) | 31.0 (27.5, 36.3) | 57.5 (50.1, 64.2) | 76.0 (66.7, 83.0) | 19.4 (16.1, 22.4) | 37.7 (34.0, 42.1) | 53.5 (46.8, 59.8) | 59.2 (50.7, 67.2) |
| AN | 95 777 | 106 886 | 56 509 | 35 563 | 144 378 | 134 660 | 61 922 | 34 719 |
| Diabetes | | | | | | | | |
| OR (95 % CI) | 1.7 (1.5, 1.9) | 3.3 (2.8, 4.0) | 6.4 (5.0, 8.3) | 10.2 (6.5, 15.8) | 2.0 (1.8, 2.4) | 4.1 (3.4, 4.8) | 6.8 (5.3, 8.7) | 8.4 (6.0, 11.9) |
| PAR % (95% CI) | 3.7 (2.7, 4.7) | 18.5 (15.1, 22.9) | 45.2 (38.0, 52.8) | 67.9 (55.8, 77.3) | 5.7 (4.6, 7.7) | 25.4 (20.9, 29.5) | 47.0 (39.7, 54.1) | 56.3 (46.5, 65.5) |
| AN | 9 767 | 26 823 | 21 616 | 17 477 | 10 517 | 32 857 | 24 580 | 16 245 |
| Heart Disease | | | | | | | | |
| OR (95% CI) | 1.1 (0.97, 1.3) | 1.4 (1.2, 1.7) | 1.8 (1.4, 2.4) | 2.2 (0.98, 4.9) | 1.0 (0.9, 1.2) | 1.4 (1.2, 1.6) | 1.1 (0.8, 1.4) | 1.7 (1.1, 2.5) |
| PAR % (95% CI) | 0.6 (0, 1.8) | 3.1 (1.6, 5.3) | 6.8 (3.5, 11.3) | 11.8 (0, 30.2) | 0 (0, 1.2) | 3.0 (1.5, 4.5) | 0.6 (0, 2.2) | 5.2 (0.8, 10.5) |
| AN | 1 803 | 3 622 | 1 946 | 1 468 | 0 | 2 760 | 114 | 673 |

+ Logistic regression model was used for each obese category, so PAR was additive.

* Bootstrap estimates were used for 95% CIs.

† AN: attributable number.

## INTERPRETATION

Using a cross-sectional study design to focus on prevalence, we provided current estimates of the burden of obesity by levels and of the contribution of comorbidity in the Canadian population.

The overall prevalence of obesity in Canada has increased at least from 1970 onwards[5] and it is consistent with the notion of a worldwide obesity epidemic[14]. Although obesity has increased greatly in Canada, we observed that its prevalence ranks in the middle of the pack internationally and is only approximately half the rate in the U.S.[17,18,19]. We also found elevated obesity with increased age, but it was less common in persons aged 65 or over among both sexes. The finding possibly was due to progressive

**TABLE 5**
**ORs* for Obesity Associated with Cluster of Risk Factors† by Sex, CCHS 2003, Canada**

| Sex | Cluster of Risk Factors† | BMI Category | | | | |
|---|---|---|---|---|---|---|
| | | 18.5–24.9 | 25.0–29.9 | 30.0–34.9 | 35.0–39.9 | ≥40 |
| Male | No | 6 658 | 7 498 | 2 208 | 452 | 123 |
| | Yes | 295 | 256 | 103 | 36 | 17 |
| | OR (95% CI) | 1 | 0.7 (0.5, 1.0) | 1.5 (0.9, 2.4) | 2.2 (1.1, 4.6) | 2.0 (0.5, 8.3) |
| Female | No | 10 520 | 5 096 | 1 783 | 500 | 214 |
| | Yes | 720 | 640 | 334 | 116 | 72 |
| | OR (95% CI) | 1 | 1.6 (1.3, 2.1) | 3.9 (2.8, 5.5) | 4.9 (2.9, 8.3) | 4.9 (2.3, 10.5) |

* ORs adjusted for age, race, language spoken, location, marital status, smoking status, and alcohol drinking.

† Cluster of risk factors: low education level, infrequent exercise, and low household income.

decline in BMI with aging or to excess mortality associated with increasing BMI in the presence of increasing age[20].

We found that, overall, 1.0% of men and 1.4% of women were in the obesity III category, consistent with another Canadian study[21]. Comparing our results with data from other countries, we saw that the prevalence of obesity III in men in Canada was higher than in Britain (0.8%), but lower than in the U.S. (1.5%), yet the prevalence in women was much lower than in Britain (2.6%) and the U.S. (2.8%)[17,19]. This means that, although the actual prevalence of obesity III in Canada was higher in women than in men, the relative prevalence in Canada was higher in men. Furthermore, our study indicated that the highest prevalence of obesity among different races in Canada was in the Aboriginal population. In this study, we identified BMI levels according to the WHO's guideline. The cut-offs for overweight and obesity levels do not perfectly fit each race. In New Zealand analyses[22,23], they have pointed out that use of the universal BMI cut-offs definition for overweight and obese might not be appropriate for comparison of obesity prevalence among different ethnic groups since it did not take into account body composition differences.

Our results confirm other research showing that obesity has been significantly associated with diabetes, high blood pressure, arthritis, asthma, and heart disease[7,16]. Compared with adults with normal BMI, men and women in the clinically obese category (BMI $\geq$ 40) had respective ORs for diagnosed heart disease of 2.2 and 1.7, and respective PARs of 11.8 and 5.2%, which are much smaller values than those observed for diagnosed diabetes or high blood pressure. Two reasons may account for this difference: (1) inappropriate controlling for blood pressure, diabetes, and other risk factors, or for the confounding effect of cigarette smoking on weight[7]; and (2) the absence of an accurate definition of heart disease. Further studies are needed to explore this area.

Most of the risk factors we identified for overweight and obesity were similar to variables noted in other studies, such as increasing age, low education, physical inactivity, race, language spoken most often at home, income, and smoking status[24,25]. Overall, there were fewer ORs with statistical significance in the obesity II or III category than in the overweight or obesity I category, especially among men. The reason may be related to genetic factors that could probably explain approximately 30% of the observed obesity[26,27,28].

Because risk factors often cluster, an estimate of a "single" contribution to overweight and obesity may not be the most useful statistic for intervention programs. Since most weight-reduction programs stress the importance of increased physical activity along with reduced caloric intake, risk values associated with a constellation of factors may better indicate what can be accomplished through intervention than risk estimates for a specific factor alone. We observed that the ORs for a cluster of risk factors (low education, infrequent exercise, and low household income) associated with obesity (including overweight and the three obesity levels) increased with BMI level, but remained stable between obesity II and III levels. The trend was more significant in women than in men. This implies that the cluster of risk factors had an impact on participants in BMI groups from overweight to obesity II, but not on those with obesity III, where the genetic factor may be more important, especially in women. Once again, further study should be undertaken to investigate this trend more thoroughly to identify if it is due simply to small numbers and thus the inability to find a statistically significant association at particular obesity levels.

A cross-sectional study usually suffers from indecipherable causal relationships — the disease studied and/or its treatments may change exposure levels for certain risk factors in subpopulations with different characteristics. Cross-sectional analysis cannot adjust for these causal relationships, but it can give a current picture of the considerable level of comorbidity associated with the risk factors. Another constraint of the cross-sectional approach is the reduced value of risk measures as compared with a longitudinal study. For instance, in the case of diabetes mellitus, the estimated risk is approximately twofold higher in prospective cohort studies. Consequently, the PAR estimated in a cross-sectional study likely underrepresents the actual risk of developing the condition and should be used to help understand the level of comorbidity in populations. In addition, the effect of changing exposure levels for a study variable on the gradual increase of comorbidities cannot be assessed with a cross-sectional study design.

The main limitation of this study is that weight and height were self-reported[13]. Survey participants tend to overestimate their height and underestimate their actual BMI[29,30]. Therefore, our prevalence estimates in this study are likely underestimated. Although self-reported data are highly correlated with measured data, using the former type may yield some misclassification. Based on survey data using actual body measurements collected from 35,000 people, 23% of adult Canadians (both sexes) and 8% of children aged 2–17 were obese in 2004[31]. Those findings may indicate that obesity prevalence in this study is indeed underestimated, especially in women, as suggested by another study[32].

The measure of physical activity used may dilute its true association with weight status. Physical activities performed at the office, at school, or in the home are not included in the calculations, which may lead to an underestimate of the actual amount of activity performed by some participants[33]. More comprehensive measurement of physical activity, use of objective measures, and measurement of sedentary behavior would have provided more complete assessment of the association between physical activity and excess weight.

In this study, we first analyzed the prevalence of overweight and different levels of obesity, and found that their correlates differ according to race, age, sex, urban/rural location, marital status, education level, physical activity, household income, and smoking status. The adjusted odds of having the major chronic diseases increased with increasing BMI levels. We also found that obesity levels were significantly associated with the combination of low education, infrequent exercise, and low household income, especially in women. Both obesity and obesity-related chronic diseases are preventable. Therefore, it is imperative that public health organizations continue to stress and develop more effective strategies aimed at changes in lifestyle and environmental factors; it is more urgent for people with low education, infrequent exercise, and low household income; and obesity control strategies should be obesity level specific. In addition, since clinically obese patients may become more common in Canada, practitioners have to prepare to treat them on a regular basis.
